# A Framework for Conducting Meta-analysis Studies; Methodological Concerns and Recommendations

**Published:** 2018-05

**Authors:** Aidin ARYANKHESAL, Meysam BEHZADIFAR, Nicola LUIGI BRAGAZZI, Ahmad GHASHGHAEE, Masoud BEHZADIFAR

**Affiliations:** 1. Dept. of Health Services Management, School of Health Management and Information Sciences, Iran University of Medical Sciences, Tehran, Iran; 2. Dept. of Epidemiology, Faculty of Health and Nutrition, Lorestan University of Medical Sciences, Khorramabad, Iran; 3. Dept. of Health Sciences (DISSAL), School of Public Health, University of Genoa, Genoa, Italy; 4. Health Management and Economics Research Center, Iran University of Medical Sciences, Tehran, Iran

## Dear Editor-in-Chief

Systematic reviews of medical evidence count as important part of research due to their robustness and credibility and wide range of audience. Systematic reviews usually aim to answer a question when there is no clear answer straightforward to make a decision based on, or the answers are controversial. Hence, the questioners, who may be same of authors or may not, make their best to accumulate all present evidence to reach a consensus so that results in evidence-based clinical decision-making or evidence-informed policymaking.

Meta-analysis is a statistical technique that helps to aggregate findings from a systematic review. In other words, meta-analysis helps to combine small samples of single studies into a compound study with a much bigger sample size. Such bigger sample size leads to much narrower confidence interval around the mean and estimation that is so more precise is obtained.

Researchers in many countries around the world conduct systematic reviews and publish the results in scientific journals which are among the main sources of evidence for policy and decision making. Such studies, especially ones with meta-analyses, should be resulted from robust methodologies and careful investigation of researchers otherwise may end with misleading information for whom use them for policy and decision-making. We summarize a framework, based on scientific and logical structure, for conducting meta-analyses as follows:

- Not all systematic reviews end with meta-analysis. If the interested data are not numbers, we cannot usually do meta-analysis. Moreover, some numeric information, such as prevalence of a disease across different provinces of a country, should not be accumulated if they are not comprehensive and exclusive or are related to different years. There is more consideration for conducting systematic reviews discussed through next points.
- In all studies, meta-analysis should come from appropriate keywords, sensitive and specific search strategy, comprehensive search in related scientific databases and unpublished works (1).
- Meta-analysis should be conducted on studies selected based on precisely defined inclusion and exclusion criteria. The criteria should be reported clearly to the readers (2).
- Studies selected for meta-analysis should pass certain quality criteria. Measuring the quality of the selected studies is very important. The main purpose of this section is generally to investigate the reliability and validity of the selected studies. Certain tools are available to assess the quality of any kind of study. STROBE, CONSORT, CASP, JADAD, and MOOSE are among frequently used ones (3).
- Data extraction from the selected studies for the final analysis step should be explained as a complete process. The target data, standard forms for data extraction, number of reviewers who extract data parallelly and independently (to make sure that the extracted data are valid), and the consensus manner if the reviewers disagreed (4).
- Statistical analysis section is one of the important parts that explained clearly and accurately. First, the appropriate statistical method should be determined in terms of the type of model- random or fixed effect- and the rationale behind the selection. Any type of indicator including prevalence, relative risk, risk difference, odds ratio, mean difference, standard mean difference can be accumulated into a comprehensive mean (5). Confidence intervals and *P*-values also should be reported where a mean is reported. However, heterogeneity of target indicators should be investigated, so that if data are heterogeneous statistically, aggregating them into a weighted mean should be avoided. Sometimes sub-group analysis can solve the problem of heterogeneity. To examine the heterogeneity Chi2 and I2, or Galbraith and Labbe plots can be used (6).
- To examine the changes of target indicator over time cumulative meta-analysis can be which needs to sort the target measures chronologically from the oldest to the newest.
- One of main issues in meta-analysis is matter of outliers. If the results of one or a few of studies are significantly different than others’, there is sufficient justification to re-check the quality of study. Sensitivity analysis can resolve this issue as well (7).
- Finally to examine the publication bias Begg’s or Egger’s tests and funnel plot can be used (8).
